# Sub-threshold depressive symptoms and brain structure: A magnetic resonance imaging study within the Whitehall II cohort^☆^

**DOI:** 10.1016/j.jad.2016.06.049

**Published:** 2016-11-01

**Authors:** Charlotte L. Allan, Claire E. Sexton, Nicola Filippini, Anya Topiwala, Abda Mahmood, Enikő Zsoldos, Archana Singh-Manoux, Martin J. Shipley, Mika Kivimaki, Clare E. Mackay, Klaus P. Ebmeier

**Affiliations:** Department of Psychiatry, University of Oxford, Warneford Hospital, OX3 7JX, United Kingdom

**Keywords:** Brain, Grey matter, White matter, Depression, Magnetic resonance imaging

## Abstract

**Background:**

Late-life sub-threshold depressive symptoms (i.e. depressive symptoms that do not meet the criteria for a diagnosis of major depressive disorder) are associated with impaired physical health and function, and increased risk of major depressive disorder. Magnetic resonance imaging (MRI) studies examining late-life major depressive disorder find structural brain changes in grey and white matter. However, the extent to which late-life sub-threshold depression is associated with similar hallmarks is not well established.

**Methods:**

Participants with no history of major depressive disorder were selected from the Whitehall Imaging Sub-Study (n=358, mean age 69±5 years, 17% female). Depressive symptoms were measured using the Centre for Epidemiological Studies Depression Scale (CES-D) at three previous Whitehall II Study phases (2003–04, 2007–09 and 2012–13) and at the time of the MRI scan (2012–14). The relationships between current and cumulative depressive symptoms and MRI brain measures were explored using Voxel-Based Morphometry (VBM) for grey matter and Tract Based Spatial Statistics (TBSS) for white matter.

**Results:**

Current sub-threshold depressive symptoms were associated with significant reductions in fractional anisotropy and increases in axial and radial diffusivity. There were no significant relationships between current depressive symptoms and grey matter measures, or cumulative depressive symptoms and MRI measures.

**Limitations:**

The prevalence (10%) of sub-threshold depressive symptoms means that analyses may be underpowered to detect subtle differences in brain structure.

**Conclusions:**

Current sub-threshold depressive symptoms are associated with changes in white matter microstructure, indicating that even mild depressive symptoms are associated with similar MRI hallmarks to those in major depressive disorder.

## Highlights

• This study investigated sub-threshold depressive symptoms in a community sample.
• Depressive symptoms were measured 4 times over 11 years.
• Brain MRI variables examined grey matter volume and white matter microstructure.
• Current depressive symptoms were associated with reduced white matter integrity.
• Sub-threshold symptoms show similar MRI hallmarks to major depressive disorder.

## 1 Introduction

Case-control magnetic resonance imaging (MRI) studies of late-life major depressive disorder have repeatedly documented reductions in grey matter volumes in the orbitofrontal cortex, hippocampus, putamen and thalamus (Du et al., 2014, Sexton et al., 2013), and reduced white matter integrity in frontal and temporal tracts (Sexton et al., 2009, Shimony et al., 2009, Wen et al., 2014). Furthermore, within clinical samples, there is evidence to support a continuum of structural brain changes associated with increasing severity and duration of symptoms (Bell-McGinty et al., 2002, Nobuhara et al., 2006).

Sub-threshold depressive symptoms (i.e. depressive symptoms that do not meet the criteria for a diagnosis of major depressive disorder) are reported in approximately 10% of older adults in community settings, and are associated with similar functional and medical comorbidities to those found in major depressive disorder (Lyness et al., 2007, Meeks et al., 2011). They are associated with an increased risk for developing major depressive disorder. MRI studies suggest that similar to late-life major depressive disorder, late-life sub-threshold depression may also be associated with reductions in grey matter volumes and white matter integrity (Dotson et al., 2009, Geerlings et al., 2012; Hayakawa et al., 2013; Lamar et al., 2010; Tudorascu et al., 2014). However, these studies vary in the approach used and the results reported to date. If relationships between MRI measures and sub-threshold symptoms can be replicated and confirmed, this could have an important public health impact, suggesting that sub-threshold depressive symptoms may warrant greater clinical attention.

We investigated the relationship between sub-threshold depressive symptoms, and MRI measures in a UK occupational-based cohort. Current depressive symptoms were measured at the time of the MRI scan, and long-term depressive symptoms were measured at four time points spanning a 10 year period. We hypothesised that sub-threshold depressive symptoms would be associated with the hallmarks of major depressive disorder, and that current and cumulative depressive symptoms would be associated with reduced grey matter volumes and reduced white matter integrity in frontal-subcortical regions.

## 2 Methods

### 2.1 Participants

The sample was drawn from 534 participants recruited to take part in the Whitehall II Imaging Sub-Study between May 2012 and December 2014 (Filippini et al., 2014). All participants were members of the Whitehall II Study, a prospective occupational cohort study established in 1985 to investigate the social gradient in health and disease (Marmot and Brunner, 2005). All civil servants aged 35–55 working in the London offices of 20 Whitehall departments in 1985–1988 were invited to participate. The response rate was 73% and a sample of 10,308 people (6895 men, 3413 women) was recruited. These participants were employed in a wide variety of roles from clerical work, through to senior administration grades (salaries ranging from £7387 to £87,620), reflecting a diverse social gradient as seen in the general population (Marmot and Brunner, 2005). Extensive data were collected through face-to-face contact with all participants at phases 1 (1985–1988), 3 (1991–1993), 5 (1997–1999), 7 (2003–2004), 9 (2007–2009) and 11 (2012–2013); intervening phases consisted of postal questionnaires (Phases 2, 4, 6 and 8), or face-to-face contact with a smaller proportion of the total sample (Phase 10). For the Whitehall Imaging sub-study, based at the University of Oxford, participants from phase 11 (2012–13) were selected at random from the whole Whitehall cohort and were invited to take part. Ethical approval was obtained from the University of Oxford Central University Research Ethics Committee, and the UCL Medical School Committee on the Ethics of Human Research. Informed written consent was obtained from all participants.

### 2.2 Inclusion and exclusion criteria

Eligible participants reported no history of dementia or neurological illness, did not display significant abnormalities on structural MRI scans, had no history of major depressive disorder (assessed using the Structured Clinical Interview for DSM IV mood disorders (First et al., 2002)), did not report use of anti-depressant or other psychotropic medication at assessments administered in 2003–2004, 2007–2009, 2012–2013 and 2012–2014 and had complete data relating to CES-D and MRI measures.

### 2.3 Demographics

Age, sex, education and cognitive performance were recorded for all participants. Education was scored on a five-point scale: (1) no qualifications, (2) O levels or equivalent, (3) A levels, college certificate or professional qualification, (4) degree, (5) higher degree (Singh-Manoux et al., 2005). Cognition was assessed at the time of the MRI scan using the Montreal Cognitive Assessment (MoCA, (Nasreddine et al., 2005)).

### 2.4 Assessment of depressive symptoms

Depressive symptoms were assessed using the Centre for Epidemiological Studies Depression Scale (CES-D), a clinically validated self-report questionnaire (Radloff, 1977).

Current depressive symptoms were measured at the time of the MRI scan (2012–2014). First, CES-D score was used to dichotomise participants into control and sub-threshold depression groups, with a score greater than 10 used to define sub-threshold depression (as in Tudorascu et al. (2014)). Second, CES-D scores were considered as a continuous variable across all participants.

Cumulative depressive symptoms reflected scores across four time-points: three previous Whitehall II Study phases (2003–2004, 2007–2009 and 2012–2013) and the time of the MRI scan (2012–2014). First, the number of times participants had been classified as displaying sub-threshold depressive symptoms was calculated. Second, the mean CES-D score across four time-points was examined.

### 2.5 Assessment of Framingham Stroke Risk Profile

Framingham Stroke Risk Profile (FSRP) was calculated for all participants based upon the following predictors: age, systolic blood pressure, diabetes mellitus, cigarette smoking, prior cardiovascular disease, atrial fibrillation, left ventricular hypertrophy and use of hypertensive medication (D'Agostino et al., 1994).

### 2.6 MRI acquisition and analysis

MRI data were acquired at the Oxford Centre for Functional MRI of the Brain (FMRIB) using a 3-Tesla, Siemens Magnetrom Verio scanner with 32-channel head coil. Image analysis was performed using tools from the FMRIB Software Library (FSL, version 5.0; http://www.fmrib.ox.ac.uk/fsl).

#### 2.6.1 Tissue types

Partial-volume tissue segmentation was performed using the FMRIB Automated Segmentation Tool (FAST) (Zhang et al., 2001). Whole brain volume was obtained by summing the volumes of grey matter, white matter and cerebrospinal fluid (CSF), and grey matter, white matter and CSF percentages were calculated.

#### 2.6.2 Grey matter volume

T1-weighted structural images were acquired using a three-dimensional rapid gradient echo sequence with repetition time 2530 ms, echo time 7.37 ms, flip angle 7°, field of view 256 mm and voxel dimensions 1.0 mm isotropic. T1-weighted images were processed using fsl_anat (http://fsl.fmrib.ox.ac.uk/fsl/fslwiki/fsl_anat).

Voxel-based morphometry (VBM) was carried out using FSL-VBM (Douaud et al., 2007), an optimised VBM protocol (Good et al., 2001) that uses FSL tools (Smith et al., 2004). Grey matter was segmented from brain extracted structural images, before being registered to MNI 152 standard space using non-linear registration (Andersson et al., 2007a, Andersson et al., 2007b). These images were averaged and flipped along the x-axis to create a left-right symmetric, study-specific grey matter template. Next, all native grey matter images were non-linearly registered to the study specific template and modulated to correct for local expansion (or contraction) due to the non-linear component of the spatial transformation. The modulated grey matter images were then smoothed with an isotropic Gaussian kernel with sigma of 3 mm.

#### 2.6.3 White matter microstructure

Diffusion Tensor Imaging (DTI) scans were acquired with an echoplanar imaging sequence (60 diffusion weighted directions, b-value 1500 s/mm^2^; 5 non-diffusion weighted images, b-value 0 s/mm^2^, with one b0 volume acquired in the reversed phase encoded direction) with repetition time 8900 ms, echo time 91.2 ms, field of view 192 mm and voxel dimensions 2.0 mm isotropic. Corrections for head motion as well as susceptibility and eddy-current induced distortions were performed using the FSL tool *eddy* (Andersson and Sotiropoulos, 2016). This employs a generative model approach to estimate all types of distortion, coupled with a single resampling step with spline interpolation to correct for all distortions simultaneously. Slices were classified as outliers and replaced if the signal was found to be more than three standard deviations from the Gaussian process predicted slice. If over 10 slices were identified as outliers within a volume, the volume was removed. If more than five volumes were removed, than the scan was excluded from analyses.

Voxelwise statistical analysis of fractional anisotropy (FA), axial diffusivity (AD) and radial diffusivity (RD) data was carried out using Tract Based Spatial Statistics (TBSS) (Smith et al., 2006), part of FSL (Smith et al., 2004). FA maps were created by fitting a tensor model to the raw diffusion data using DTIFit, part of FMRIB's Diffusion Toolbox (http://fsl.fmrib.ox.ac.uk/fsl/fdt). This fits a diffusion tensor model to the raw diffusion data and then brain-extracts using BET (Smith, 2002). All participants' FA data were aligned into a common space using FMRIB's Nonlinear Registration Tool (FNIRT) (Andersson et al., 2007a, Andersson et al., 2007b), which uses a b-spline representation of the registration warp field (Rueckert et al., 1999). Next, the mean FA image was created and thinned to create a mean FA skeleton representing the centres of all tracts common to the group. This method was repeated for mean diffusivity (MD), axial diffusivity (AD) and radial diffusivity (RD).

### 2.7 Statistical analysis

We employed permutation-based methods for non-parametric testing for all analyses (Winkler et al., 2014), using Randomise for voxelwise analysis of MRI data (5000 permutations), and Permutation Analysis of Linear Models (PALM) for all other analyses.

For analyses of current sub-threshold depressive symptoms, group differences in demographic and MRI measures were assessed. Next, linear associations between current CES-D score and MRI measures were examined. For analyses of cumulative sub-threshold depressive symptoms, linear associations between the number of times participants had been classified as displaying sub-threshold depressive symptoms and demographic and MRI measures were assessed. Next, associations with the average CES-D score over all four time-points were examined. Age, sex, education and MoCA score were included as covariates in all analyses examining MRI data, with significant analyses repeated with FSRP as an additional covariate. In all voxel-wise analyses, the significance threshold was set at p<0.05 using the threshold-free cluster enhancement option.

## 3 Results

**Fig. 1:**
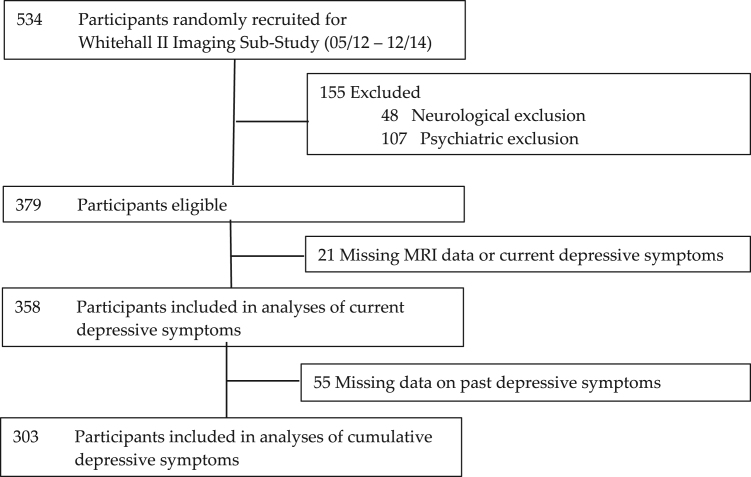
Attrition of participants.

Of 534 participants recruited for the Whitehall II Imaging Sub-Study (2012–2014), 358 were included in analyses of current depressive symptoms, and 303 in analysis of cumulative depressive symptoms (Fig. 1). Participants excluded because of missing data were not significantly different to the included sample in age or sex, but displayed significantly lower education levels and MoCA scores compared with participants with full datasets (Supplementary material, Table S1). Mean CES-D scores were 4.1±4.9 for current symptoms, and 5.1±4.2 for cumulative symptoms (Supplementary material, Figs. S1 and S2 display histograms showing the spread of CES-D scores).

### 3.1 Current depressive symptoms

35 participants (10%) had CES-D scores >10 at the time of the MRI assessment (Table 1). Participants with scores >10 did not display significant differences in age, sex, education, MoCA scores or FSRP compared to those with scores ≤10. Grey matter, white matter and CSF percentages, were not significantly different between groups, and no significant differences in grey matter volume were detected locally using FSL-VBM.

**Fig. 2:**
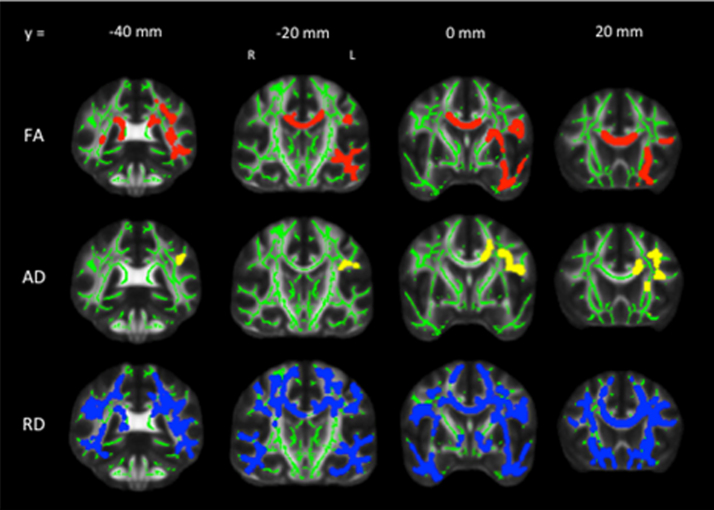
Localisation of group differences in DTI measures between CES-D≤10 and CES-D>10 groups. Voxels displaying a significant reduction in FA (red), increase in AD (yellow) and increase in RD (blue) in the sub-threshold depression group, dilated for illustrative purposes, are overlaid on a green skeleton. Age, sex, education and MoCA scores were included as covariates. (For interpretation of the references to color in this figure legend, the reader is referred to the web version of this article.)

Analyses of global DTI metrics showed that sub-threshold depressive symptoms were associated with lower global FA, and higher global AD and RD. In voxel-wise analyses, 10% of all voxels displayed a significant reduction in FA, 3% of all voxels displayed a significant increase in AD, and 34% of all voxels displayed a significant increase in RD. Significant voxels primarily fell within the frontal lobe, with parietal and temporal lobes also affected (Table 2). The localisation of significant effects is illustrated in Fig. 2, illustrating that significant voxels particularly fell within the corpus callosum, and the inferior and superior longitudinal fasciculi. Results remained significant after FSRP was included as an additional covariate in analysis of global DTI metrics (FA p=0.020, AD p=0.006, RD p=0.007) and in voxel-wise analyses (FA 10% voxels significant, AD 2% of voxels significant, RD 35% of voxels significant).

CES-D score at the time of the MRI assessment was also examined as a continuous variable, and was not significantly associated with age, sex, education, MoCA score, FSRP or global MRI measures (Supplementary material, Table S2). No significant associations were detected between current CES-D score and voxel-wise measures of grey matter volume, using FSL-VBM, or white matter microstructure, using TBSS.

### 3.2 Cumulative depressive symptoms

The number of times participants had a CES-D score >10 over four time-points was examined. 200 participants (66%) had scores ≤10 at all four time-points, 64 (21%) had a score of >10 at a single time-point, 17 (6%) had a score of >10 at two time-points, 11 (4%) had a score of >10 at three time-points, and 10 (4%) had scores >10 at all four time-points (Supplementary material, Table S3). There were no significant associations between the number of times participants scored >10 on the CES-D scale and age, sex, education or FSRP. The number of times a participant scored >10 was significantly negatively associated with the MoCA score.

The number of times participants scored >10 on the CES-D scale was not significantly associated with the percentage of grey matter, white matter or CSF, or global FA, AD or RD. Voxel-wise analyses of grey matter volume, using FSL-VBM, or white matter microstructure, using TBSS, similarly yielded no significant results.

Mean CES-D score over four time-points was examined as a continuous variable (Supplementary material, Table S4). The mean CES-D score was not associated with age, sex, education or FSRP, but was significantly negatively associated with MoCA score. Analyses of grey matter percentage and CSF percentage were not significant, but percentage white matter was positively associated with mean CES-D score. No significant associations were detected between mean CES-D score and global DTI measures, or with voxel-wise analyses of grey matter.

## 4 Discussion

In a prospective cohort sub-study of over 300 participants, we found that current sub-threshold depressive symptoms were associated with a pattern of reduced FA and increased AD and RD, particularly within the corpus callosum and the inferior and superior longitudinal fasciculi. This indicates that even mild depressive symptoms are associated with some of the same MRI hallmarks as major depressive disorder. We did not find an association with cumulative CES-D scores, suggesting that sub-threshold depressive symptoms do not have cumulative effects on the brain. Rather, it was depressive symptoms at the time of the MRI scan that were relevant to white matter structural changes. This may indicate the importance of *current* depressive symptoms, or *late-life* symptoms, since previous studies of major depressive disorder have shown DTI measures to be associated with later age at onset (Sexton et al., 2012b).

Our findings are consistent with studies that identified an association between sub-threshold depressive symptoms and reduced white matter integrity (Dotson et al., 2009, Lamar et al., 2010, Shimony et al., 2009, Tudorascu et al., 2014). These studies identified altered white matter integrity in frontal regions (Dotson et al., 2009, Lamar et al., 2010), within the anterior cingulum bundle (Shimony et al., 2009) and with global changes in FA (Tudorascu et al., 2014). A meta-analysis of DTI studies in late-life depression found consistent reductions in white matter integrity within the frontal lobe, corpus callosum, uncinate fasciculi and cingulum (Wen et al., 2014). While our results do not mirror these previous studies exactly, there is significant overlap between our findings and previous studies of sub-threshold depressive symptoms and late-life depressive disorder that find altered white matter microstructure in frontal-subcortical regions.

Based on previous literature, the most likely explanation for an association between white matter micro-structural changes and depressive symptoms is the presence of vascular risk factors. The vascular depression hypothesis proposes that vascular risk factors can predispose, precipitate or perpetuate late-onset depression (Alexopoulos et al., 1997). Vascular risk factors, as measured by the FSRP have been shown to correlate with DTI metrics in late-life major depressive disorder (Allan et al., 2012). In the current study FSRP was not significantly associated with depressive symptoms, and differences in DTI metrics between current sub-threshold depression and control groups remained significant after including FSRP as an additional covariate i.e. our results do not provide additional support for the vascular depression hypothesis. Other factors, for example inflammatory changes may provide an alternative explanation for the association (Valkanova et al., 2013).

Caution is needed in the interpretation of our findings, given that there were no significant associations between current depressive symptoms and grey matter changes, in contrast with some previous studies (Dotson et al., 2009, Geerlings et al., 2012, Shimony et al., 2009, Tudorascu et al., 2014). Several studies highlighted an association between sub-threshold depressive symptoms and volume reductions in the anterior cingulate cortex (Dotson et al., 2009, Shimony et al., 2009, Tudorascu et al., 2014). However, reductions in grey matter volume in other regions were only identified in single studies: orbitofrontal cortex (Dotson et al., 2009), hippocampus (Geerlings et al., 2012), right rectal gyrus (Shimony et al., 2009) and insula (Tudorascu et al., 2014). The inconsistency of results in studies of sub-threshold depressive symptoms, as well as in late-life depressive disorder (Du et al., 2014) mean that our lack of positive findings are not surprising. Few studies examined both grey and white matter changes in the same study, and our findings of white matter changes without grey matter changes are consistent with our previous study of late-life major depressive disorder (Sexton et al., 2012a).

Given that our study included a larger sample size than many previous studies (Dotson et al., 2009, Lamar et al., 2010, Shimony et al., 2009), it is unlikely that our null findings are not simply an issue of power. Rather, our results may differ from previous studies due to differences in inclusion and exclusion criteria, and the demographics of our sample. For example, in order to exclusively examine sub-threshold depressive symptoms, we excluded participants with a history of major depressive disorder. We also excluded participants using anti-depressant medication as this was considered a marker of depression severity and an additional confounding factor that could influence structural brain measures (Geerlings et al., 2012). Positive results in previous studies in which participants with major depressive disorder were included may be driven by structural brain changes in these participants.

The previous literature is inconsistent about the precise definitions of sub-threshold depression and an important consideration in interpreting our results is that the average CES-D score is relatively low compared with previous studies (e.g. mean CES-D 4.1 in this study, compared to 7.0 (Tudorascu et al., 2014)). Although the proportion of participants in this sample with a CESD score >10 (10%) mirrors expected rates of sub-threshold depressive symptoms (Meeks et al., 2011) the relatively low burden of depressive symptoms overall may mean that our analyses are underpowered to detect subtle differences in brain structure, contributing to some of our null findings. Finally, the generalizability of our results may be reduced by a sample of participants who were predominantly white, male and with above average educational attainment.

Despite these limitations, the strengths of our study include a large sample size from an established cohort, identification of depressive symptoms cross-sectionally and longitudinally, and the MRI acquisition and analysis methods employed. Our inclusion and exclusion criteria allowed us to be confident of exclusively examining sub-threshold depressive symptoms.

In summary, sub-threshold depressive symptoms have been associated with poorer functioning and physical health (Lyness et al., 2007), similar to impairments associated with major depressive disorder. In this study we found that sub-threshold depressive symptoms were associated with impaired white matter microstructure, overlapping with the hallmarks that would be expected in major depressive disorder. We did not find any significant associations with grey matter. Future research should explore the optimal CES-D cut off that can reliably detect neuroanatomical changes associated with depressive symptoms, and should consider whether changes in MRI brain measures may be a predisposing factor in sub-threshold depression.

## Author disclosure

None.

## Contributors

KPE, KM, CEM, ASM and MJS designed the study and wrote the protocol. AM and EZ recruited participants; AM, EZ, CLA and AT undertook participant interviews. NF undertook MRI pre-processing and assisted with early analyses of depressive symptoms. CES managed analyses and contributed to the first draft. CLA wrote the first draft. All authors contributed to and have approved the final manuscript. CLA and CES contributed equally to the manuscript and are listed as joint first authors.

## Role of the funding source

The Whitehall Imaging sub-study is funded by a MRC Lifelong Health and Wellbeing’ Program Grant (UK Medical Research Council: G1001354), the Gordon Edward Small's Charitable Trust (SC008962), and the HDH Wills 1965 Charitable Trust (PI: KPE). C. L. A. is supported by the National Institute of Health Research, Wolfson College, Oxford and Oxford University Clinical Academic Graduate School. C. E. S and C. E. M are supported by the National Institute for Health Research (NIHR) Oxford Biomedical Research Centre based at Oxford University Hospitals NHS Trust and the University of Oxford. N. F. and A. M. were funded by the HDH Wills 1965 Charitable Trust. A. S.-M. is supported by the National Institute on Aging, NIH (R01AG013196; R01AG034454). M. K. is supported by the UK Medical Research Council (K013351), the US National Institutes of Health (R01HL036310; R01AG034454) and an ESRC professorship. K. P. E. is supported by UK Medical Research Council (G1001354), the Gordon Edward Small's Charitable Trust (SC008962) and the HDH Wills 1965 Charitable Trust. The data sharing policy for Whitehall II data has been published here: http://www.ucl.ac.uk/whitehallII/data-sharing. The data sharing policy for the Whitehall II MRI substudy is subject to the same rules for merging of imaging and clinical data. The data sharing policy of the substudy itself is modelled on this and has been approved by MRC. We will publish it when the data set is coming closer to completion in 2016. There will be a period of 2 years to allow the research group and collaborators time for analysis and publication of the data.

## Conflicts of interest

The authors report no conflicts of interest.

## Figures and Tables

**Table 1:** Demographic and MRI measures in those with and without current depressive symptoms.

	CES-D≤10	CES-D>10	Cohen’s d	p
*Demographics*				
N (%)	323 (90%)	35 (10%)		
Age (years)	69.6±5.3	68.8±4.9	-0.15	0.209
Sex (N females, %)	55 (17%)	6 (17%)	<0.01	0.571
Education	3.4±1.1	3.6±1.0	0.19	0.166
MoCA	27.2±2.3	26.7±3.0	-0.21	0.138
FSRP	11.4±8.2	10.8±7.0	-0.08	0.344
				
*Depressive symptoms*				
Current CES-D	2.8±2.7	15.6±5.0	4.28	<0.001
				
*Tissue types*				
WB (cm^3^)	1442±126	1434±161	−0.06	0.365
GM (%)	38.5±1.9	38.4±2.0	−0.09	0.306
WM (%)	38.8±1.9	38.9±2.1	−0.06	0.382
CSF (%)	22.7±2.7	22.8±2.5	0.12	0.250
				
*Global DTI metrics*				
FA	0.48±0.02	0.47±0.02	−0.37	0.021
AD (x10^3^)	1.07±0.02	1.08±0.03	0.48	0.006
RD (x10^3^)	0.48±0.03	0.49±0.03	0.47	0.008

Values are mean±standard deviation. Age, sex, education and MoCA score were included as covariates in analyses of tissue types and global DTI metrics.

**Table 2:** Localisation of group differences in DTI measures between CES-D≤10 and CES-D>10 groups.

	**No of significant voxels**			**% of significant voxels**		
	**FA**	**AD**	**RD**	**FA**	**AD**	**RD**
Global	12,556	3331	44,144	10	3	34
Frontal	5206	2749	22,931	11	6	48
Parietal	3427	582	8808	13	2	33
Occipital	1368	0	2179	13	0	20
Temporal	2381	0	9915	10	0	42

Number and percentage of voxels significant at p<0.05, after correction for multiple comparisons across space, with age, sex, education and MoCA score included as covariates.

## Footnotes

Appendix A Supplementary data associated with this article can be found in the online version at doi:10.1016/j.jad.2016.06.049.
