# Supplementary material for: Sub-threshold depressive symptoms and brain structure: A magnetic resonance imaging study within the Whitehall II cohort
Source: J Affect Disord. 2016 Nov 1;204:219–25. doi: 10.1016/j.jad.2016.06.049 (PMC5022868; doi:10.1016/j.jad.2016.06.049)
Supplement: Supplementary file 1 — Supplementary material [file mmc1.docx]

**Supplementary material**

**Table S1. Comparison of participants included and those excluded due to missing data**

* corrected for multiple contrasts

|  | **Complete Data** | **Missing Data** | **Cohen’s d** | **p*** |
| --- | --- | --- | --- | --- |
| N | 303 | 76 |  |  |
| Age (years) | 69.5 ± 5.3 | 69.5 ± 5.2 | -0.01 | 0.709 |
| Sex (N females, %) | 51 (17%) | 12 (16%) | -0.03 | 0.685 |
| Education | 3.5 ± 1.1 | 3.2 ± 1.2 | **-0.28** | **0.031** |
| MoCA | 27.3 ± 2.3 | 26.7 ± 2.5 | **-0.26** | **0.040** |

**Table S2: Association between demographic and MRI measures with current CES-D as a continuous variable**

Values are mean ± standard deviation. Age, sex, education and MoCA score were included as covariates in analyses of tissue types and global DTI metrics.

|  | |  | **Pearson’s r** | **p** |
| --- | --- | --- | --- | --- |
|  | |  |  |  |
| *Demographics* |  | | | |
| N | | 358 |  |  |
| Age (years) | | 69.5 ± 5.3 | -0.04 | 0.247 |
| Sex (N females, %) | | 61 (17%) | 0.08 | 0.073 |
| Education | | 3.4 ± 1.1 | 0.08 | 0.069 |
| MoCA | | 27.2 ± 2.3 | -0.08 | 0.068 |
| FSRP | | 11.4 ± 8.1 | 0.02 | 0.325 |
|  | |  |  |  |
| *Depressive Symptoms* | | | | |
| Current CES-D | | 4.1 ± 4.9 |  |  |
|  | |  |  |  |
| *Tissue Types* | |  |  |  |
| Whole brain volume (cm^3^) | | 1141 ± 130 | <0.01 | 0.451 |
| Grey Matter (%) 2 | | 38.5 ± 1.9 | -0.04 | 0.178 |
| White Matter (%) 3 | | 38.8 ± 1.9 | 0.01 | 0.432 |
| CSF (%) 1 | | 22.7 ± 2.7 | 0.02 | 0.277 |
|  | |  |  |  |
| *Global DTI Metrics* | | | | |
| FA | | 0.48 ± 0.02 | -0.05 | 0.148 |
| AD (x10^3^) | | 1.07 ± 0.02 | 0.07 | 0.076 |
| RD (x10^3^) | | 0.49 ± 0.03 | 0.07 | 0.078 |

**Table S3: Association between demographic and MRI measures with cumulative depressive symptoms (Number of times CES-D > 10). (N=303)**

Values are mean ± standard deviation. Age, sex, education and MoCA score were included as covariates in analyses of tissue types and global DTI metrics.

|  | | **Number of Times CES-D > 10** | | | | |  |  |
| --- | --- | --- | --- | --- | --- | --- | --- | --- |
|  | | **0** | **1** | **2** | **3** | **4** | **Pearson’s r** | **p** |
|  | |  |  |  |  |  |  |  |
| *Demographics* | |  |  |  |  |  |  |  |
| N | | 200 (66%) | 64 (21%) | 17 (6%) | 11 (4%) | 11 (4%) |  |  |
| Age (years) | | 70.0 ± 5.3 | 68.8 ± 5.2 | 66.4 ± 3.0 | 71.1 ± 5.8 | 69.1 ± 6.4 | -0.08 | 0.098 |
| Sex (N females, %) | | 31 (16%) | 12 (19%) | 4 (24%) | 2 (18%) | 2 (18%) | 0.04 | 0.262 |
| Education | | 3.5 ± 1.0 | 3.3 ± 1.1 | 3.5 ± 1.1 | 3.8 ± 0.8 | 3.6 ± 1.3 | 0.02 | 0.343 |
| MoCA | | 27.6 ± 2.0 | 26.6 ± 2.8 | 27.2 ± 2.6 | 26.9 ± 2.5 | 26.8 ± 2.9 | **-0.12** | **0.023** |
| FSRP | | 11.3 ± 6.5 | 11.4 ± 8.1 | 9.5 ± 4.9 | 10.7 ± 5.1 | 8.5 ± 3.5 | -0.08 | 0.082 |
|  | |  |  |  |  |  |  |  |
| *Depressive Symptoms* | | | | | | | | |
| Mean CES-D | | 2.9 ± 1.9 | 6.6 ± 2.0 | 10.0 ± 1.9 | 13.8 ± 2.9 | 18.1 ± 3.9 | **0.88** | **<0.001** |
|  | |  |  |  |  |  |  |  |
| *Tissue Types* | |  |  |  |  |  |  |  |
| Whole Brain Volume (cm^3^) | | 1435 ± 121 | 1459 ± 156 | 1458 ± 127 | 1411 ± 81 | 1401 ± 122 | 0.01 | 0.418 |
| Grey Matter (%) | | 38.5 ± 1.9 | 38.2 ± 2.0 | 38.4 ± 1.6 | 37.9 ± 2.1 | 38.6 ± 1.6 | -0.07 | 0.097 |
| White Matter (%) | | 38.7 ± 1.8 | 39.2 ± 1.9 | 39.3 ± 2.0 | 39.4 ± 2.4 | 39.1 ± 2.1 | 0.06 | 0.132 |
| CSF (%) | | 22.8 ± 2.8 | 22.6 ± 2.7 | 22.4 ± 2.6 | 22.6 ± 3.0 | 22.3 ± 2.6 | 0.01 | 0.425 |
|  | |  |  |  |  |  |  |  |
|  | *Global DTI Metrics* | | | | | | | |
| FA | | 0.47 ± 0.02 | 0.48 ± 0.01 | 0.47 ± 0.02 | 0.48 ± 0.02 | 0.47 ± 0.02 | -0.03 | 0.318 |
| AD (x10^3^) | | 1.07 ± 0.02 | 1.07 ± 0.02 | 1.07 ± 0.02 | 1.07 ± 0.03 | 1.08 ± 0.02 | 0.06 | -0.119 |
| RD (x10^3^) | | 0.49 ± 0.03 | 0.48 ± 0.02 | 0.49 ± 0.02 | 0.48 ± 0.03 | 0.49 ± 0.03 | 0.04 | 0.214 |

**Table S4: Association between demographic and MRI measures with cumulative depressive symptoms (mean CES-D score over 4 time-points). (N=303)**

Values are mean ± standard deviation. Age, sex, education and MoCA score were included as covariates in analyses of tissue types and global DTI metrics.

|  |  | Pearson’s r | p |
| --- | --- | --- | --- |
|  |  |  |  |
| *Demographics* |  |  |  |
| Age (years) | 69.5 ± 5.3 | -0.07 | 0.120 |
| Sex (N females, %) | 51 (17%) | 0.01 | 0.388 |
| Education | 3.5 ± 1.1 | 0.07 | 0.101 |
| MoCA | 27.3 ± 2.3 | **-0.15** | **0.008** |
| FSRP | 11.1 ± 6.7 | -0.05 | 0.220 |
|  |  |  |  |
| *Depressive Symptoms* | | | |
| Mean CES-D | 5.1 ± 4.2 |  |  |
|  |  |  |  |
| *Tissue Types* |  |  |  |
| Whole Brain Volume (cm^3^) | 1439 ± 128 | 0.04 | 0.218 |
| Grey Matter (%) | 38.4 ± 1.9 | -0.07 | 0.095 |
| White Matter (%) | 38.9 ± 1.9 | **0.11** | **0.018** |
| CSF (%) | 22.7 ± 2.7 | -0.03 | 0.284 |
|  |  |  |  |
| *Global DTI Metrics* | | | |
| FA | 0.47 ± 0.02 | -0.01 | 0.406 |
| AD (x10^3^) | 1.07 ± 0.02 | 0.05 | 0.195 |
| RD (x10^3^) | 0.49 ± 0.03 | 0.03 | 0.321 |

***Abbreviations***

*MoCA Montreal Cognitive Assessment*

*FSRS Framingham Stroke Risk Profile*

**Figure S1. Histogram of current depressive symptoms**

**Figure S2. Histogram of cumulative depressive symptoms (average score across four time-points, rounded to the nearest integer)**
